# Tanshinone IIA Restores Dynamic Balance of Autophagosome/Autolysosome in Doxorubicin-Induced Cardiotoxicity via Targeting Beclin1/LAMP1

**DOI:** 10.3390/cancers11070910

**Published:** 2019-06-28

**Authors:** Xiaoping Wang, Chun Li, Qiyan Wang, Weili Li, Dongqing Guo, Xuefeng Zhang, Mingyan Shao, Xu Chen, Lin Ma, Qian Zhang, Wei Wang, Yong Wang

**Affiliations:** 1 School of Life Science, Beijing University of Chinese Medicine, Beijing 100029, China; 2 Modern Research Center for Traditional Chinese Medicine, Beijing University of Chinese Medicine, Beijing 100029, China; 3 School of Traditional Chinese Medicine, Beijing University of Chinese Medicine, Beijing 100029, China

**Keywords:** tanshinone IIA, doxorubicin, autophagic flux, Beclin1, LAMP1

## Abstract

Clinical use of the anti-cancer drug doxorubicin (DOX) is largely limited due to its severe cardiotoxicity. Dysregulation of autophagy is implicated in DOX-induced cardiotoxicity (DIC). Prior studies have indicated that Beclin1 and lysosomal-associated membrane proteins-1 (LAMP1) are critical mediators of autophagy. In this work, by assessing autophagic flux in a DOX-stimulated H9C2 model, we observed autolysosome accumulation caused by interruption of autolysosome degradation. Tanshinone IIA (TSA) is a well-known small molecule that exerts impressive cardioprotective effects on heart failure. Here, we investigated the regulation of TSA in DOX-treated zebrafish, mice, and H9C2 models. Results demonstrated that TSA remarkably improved heart function and reversed pathological changes in vivo, while TSA restored autophagic flux by promoting autolysosome degradation and autophagosome formation. Further experiments demonstrated that these effects were mediated through upregulation of Beclin1 and LAMP1. The mTOR agonist MHY1485 was shown to abrogate the effect of TSA via the UNC-51-like kinase 1 (ULK1)-Beclin1/TFEB-LAMP1 signaling pathway in vitro, demonstrating that TSA protects against DIC by promoting autophagy via the Beclin1/LAMP1 signaling pathway. We further employed a U87 model to assess whether TSA would compromise the antitumor activity of DOX. Intriguingly, the co-treatment of TSA was able to synergistically inhibit proliferative activity. Collectively, in this study we uncover the novel insight that TSA is able to reduce the cardiotoxicity of DOX without compromising antitumor activity.

## 1. Introduction

Doxorubicin (DOX) is an effective and widely used chemotherapeutic agent utilized for patients with cancer. However, its clinical use is limited due to its side effects of severe cardiotoxicity and heart failure. In particular, the symptoms of DOX-induced cardiotoxicity (DIC) can last for many years in children treated with DOX [1]. Dexrazoxane is the only cardioprotectant approved by the Food and Drug Administration (FDA) for use in DIC. However, it has been proven that dexrazoxane can induce secondary malignancies [2] and aggravate myelosuppression [3]. No matter whether these reports are solid or not, seeking novel strategies involving combinational usage of agents to minimize associated cardiotoxicity and increase chemotherapeutic efficacy should be at the forefront of cross-discipline research which combines oncology and cardiology research. Numerous studies have explored the molecular mechanisms of DIC, and several molecular elements, including DNA damage, mitochondrial damage, and the accumulation of reactive oxygen species (ROS), have been implicated in the pathogenesis of DIC [4]. However, an explicit and unifying model of the pathogenesis of DIC remains undefined.

Autophagy is a vital cell survival mechanism in which cells degrade and recycle damaged/old organelles and misfolded proteins within lysosomes to maintain cellular homeostasis [5,6]. It is becoming evident that altered autophagic activity is closely associated with cardiovascular diseases, especially DIC and DIC-caused heart failure [4]. Autophagy involves the synthesis of a double-membrane autophagosome, which is initiated by UNC-51-like kinase 1 (ULK1). Subsequently, Beclin1 (ATG6) forms a multiprotein complex with Vps34 and UVRAG and this complex is activated by ULK1 to induce autophagosome formation. Protein 1 light chain 3-I (LC3-I), an immature isoform, is lipidated and converted into mature LC3-II via conjugation with phosphatidylethanolamine (PE). P62, a ubiquitin-binding cargo receptor, engages in segregating proteins, organelles, and other aggregates into the autophagosome. Next, lysosomes fuse with the outer membranes of autophagosomes and autolysosomes are formed. In the last step of autophagy, under the mediation of lysosomal-associated membrane proteins LAMP1 and LAMP2, the autolysosome contents are then degraded by a series of lysosomal proteases [7] which autophagic flux ends. Hence, cooperation of autophagosomes and lysosomes is critical to the autophagy process, with Beclin1 being a key molecule [8]. Moreover, the mTOR kinase is a negative regulator of autophagy which exerts its biological activity through ^Thr389^p-S6K1. mTOR phosphorylates Ser757 of ULK1 to inactivate Beclin1, leading to the suppression of autophagosome formation [5]. In addition, LAMP, as the biomarker of lysosome biogenesis and function, is critical for orchestrating autophagic flux [1,9]. mTOR phosphorylates TFEB and results in the sequestration of TFEB in the cytoplasm. Studied have definitively shown that TFEB masters the transcription of target genes closely related to lysosomal biogenesis and function, including hydrolases and LAMP1, whereas impaired nuclear localization of TFEB causes the decline of lysosomal biogenesis and function [10,11]. Accumulating evidence demonstrates that the dysregulation of Beclin1/LAMP1 is involved in DIC [1,7,12]. The Beclin1/LAMP1-mediated autophagy pathway has emerged as a potential target for treating DIC. Interestingly, Beclin1, as a key scaffold for the assembly of distinct signaling complexes, also acts as a tumor suppressor protein [8].

Tanshinone IIA (TSA) is a fat-soluble component of *Salvia miltiorrhiza* and has been commonly used for the treatment of cardiovascular diseases [13,14,15,16]. Experimental studies and clinical trials have demonstrated that TSA is able to improve cardiac function and reduce apoptosis in heart failure [17,18]. In addition, recent studies have revealed that TSA is able to improve the anticancer activity of DOX and exert cardioprotective effects due to its antioxidant properties and antiapoptotic effects [19,20], suggesting that the combined treatment of TSA with DOX may be a feasible strategy to reduce DIC. Although the protective effects of TSA on DIC have been preliminarily investigated, the mechanism remains controversial. In light of the critical role that autophagy plays in DIC, the present study aims to explore whether TSA protects against DIC through the Beclin1/LAMP1 autophagy signaling pathway via in vivo and in vitro studies. We also tried to assess whether TSA could reduce the cardiotoxicity of DOX without compromising its antitumor activity.

## 2. Results

### 2.1. Effects of TSA on Cardiac Function and Structural Alterations in a DIC Mouse Model

**Figure 1:**
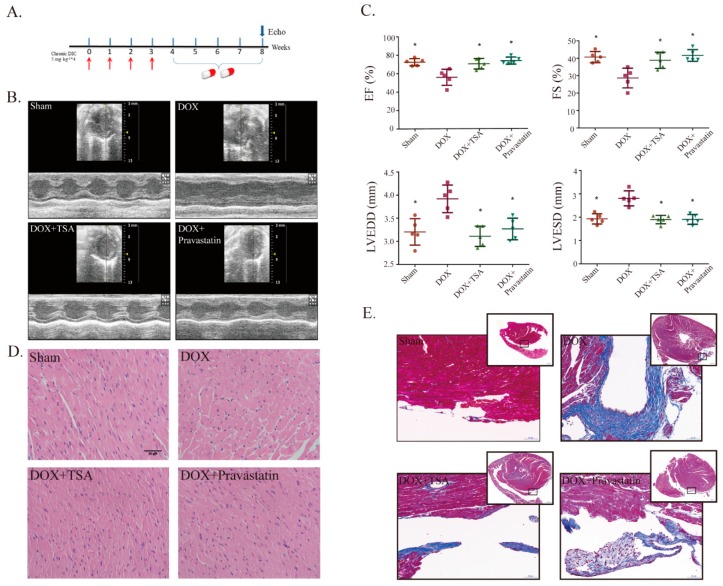
Tanshinone IIA (TSA) is shown to improve heart function and reduce pathological changes in mice. (**A**) A diagram of the experiment design in mice. (**B**) Cardiac function of mice in each group was detected by M-mode echocardiography. (**C**) Echocardiographic analysis showed that TSA can increase ejection fraction (EF) and fraction shortening (FS) values and decrease left ventricular end-diastolic dimension (LVEDD) and left ventricular end-systolic dimension (LVESD) (*n* = 5). * *p* < 0.05 is significantly different as indicated, for values in the model group. (**D**) H&E showed that TSA preserved cardiomyocyte architecture (*n* = 5). Scale bar: 20 μm. (**E**) Masson staining showed that TSA reduced collagen deposition (*n* = 5). Scale bar: 50 μm/500 μm. Legend: DOX, doxorubicin; DIC, DOX-induced cardiotoxicity. H&E, abbreviation of Hematoxylin and eosin.

A DIC animal model (Figure 1A) was generated by injecting DOX into C57BL/6 mice through the tail vein [1]. After four weeks of intragastric administration, echocardiographs showed that the DIC model had been established successfully, as evidenced by the significant decline in the ejection fraction (EF) and fraction shortening (FS) values in the model group compared with those of the sham group. In addition, left ventricular end-diastolic dimension (LVEDD) and left ventricular end-systolic dimension (LVESD) were increased in the model group, indicating that cardiac dysfunction and structural alterations appeared (Figure 1B,C). After TSA treatment, the levels of EF and FS were upregulated and LVEDD and LVESD were significantly reduced (Figure 1B,C). Hematoxylin and eosin (H&E) staining showed that the structures of cardiomyocytes were damaged in the DIC model group. DIC was associated with the disorderly arrangement of cardiac tissue, myofibrillar loss, pyknosis, and plasma-dissolved cardiomyocytes. The pathological changes were reversed significantly by TSA treatment (Figure 1D). Masson staining showed that there was a presence of fibrosis for the model group around the arterioles, but the staining of fibrosis from the sham group and TSA-treatment group was not as significant (Figure 1E). Pravastatin had similar effects to TSA (Figure 1B–E).

### 2.2. Effect of TSA on Body Weight, Serum Lactate Dehydrogenase (LDH), Creatine Kinase of Muscle–Brain Type (CKMB), and Apoptosis in the DIC Mouse Model

**Figure 2:**
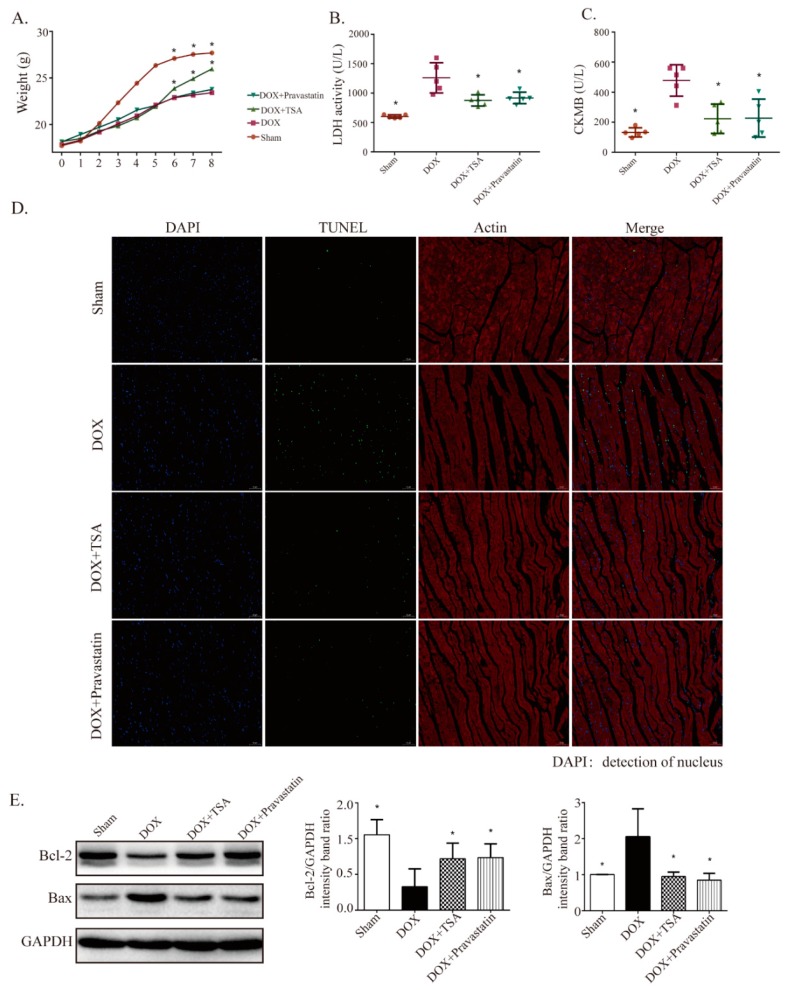
TSA is shown to increase body weight and decrease Serum LDH, creatine kinase of muscle-brain type (CKMB), and apoptosis in mice. (**A**) TSA increased body weights compared with the model group (*n* = 10). TSA decreased Serum LDH (*n* = 8) (**B**) and CKMB (*n* = 8) (**C**) compared with the model group. (**D**) TUNEL results showed that TSA was able to decrease the apoptosis rate (*n* = 5). Scale bar: 50 μm. (**E**) TSA regulated the expression of apoptosis-related proteins, including Bcl-2 and Bax, and the results showed that TSA treatment upregulated the expression of Bcl-2 and downregulated the expression of Bax (*n* = 5). All data are presented as mean ± SD. * *p* < 0.05 is significantly different as indicated, for values in the model group. LDH, abbreviation of lactate dehydrogenase; TUNEL, abbreviation of Terminal deoxynucleotidyl transferase-mediated dUTP nick-end labeling; DAPI, abbreviation of 4′,6-diamidino-2-phenylindole.

Consistent with previous results, the body weights of the mice decreased significantly for mice from the model group compared with the weights of mice from the sham group, while TSA treatment increased body weights (Figure 2A). LDH and CKMB are located in the cytoplasm of cardiomyocytes under normal conditions and the release of LDH and CKMB into the blood is a diagnostic indicator of heart failure [21]. The results showed that the levels of LDH and CKMB in the model group mice were significantly upregulated compared with those in the sham group mice, while TSA treatment reversed these changes (Figure 2B,C). Terminal deoxynucleotidyl transferase-mediated dUTP nick-end labeling (TUNEL) staining was utilized to determine the antiapoptotic effects of TSA. The apoptotic rate of cardiomyocytes in the myocardial tissue in the model group was higher than that in the sham group, while TSA treatment reduced the apoptotic rate (Figure 2D). The effects of TSA on apoptosis were further confirmed by Western blot. The results showed that the expression of Bax was upregulated and that the expression of Bcl-2 was downregulated in the model group compared to that of the sham group. TSA treatment reduced the expression of Bax and increased the expression of Bcl-2 in the DIC mouse model, exerting antiapoptotic effects (Figure 2E).

### 2.3. TSA Restores Autophagy in the DIC Mouse Model

**Figure 3:**
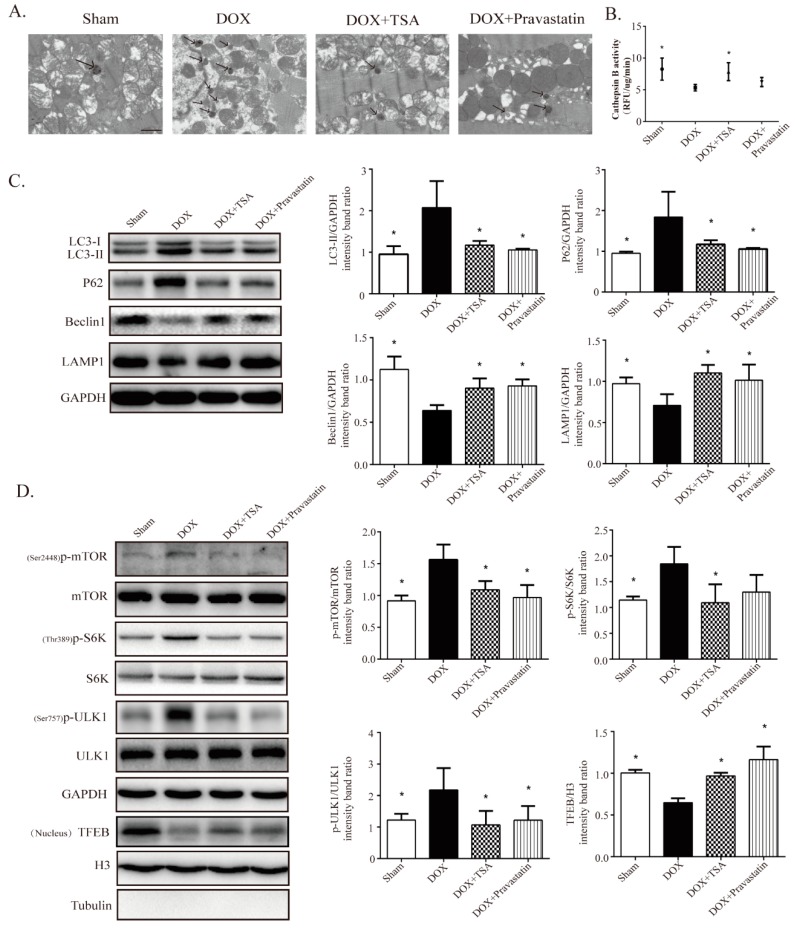
TSA is shown to restore autophagy in mice. (**A**) Representative transmission electron microscopy image of each group (*n* = 8). Scale bar: 1 μm. (**B**) TSA increased cathepsin B activity (*n* = 5). (**C**) Western blot analysis showed TSA regulated the expression of autophagy-related proteins, including LC3-II, P62, Beclin1, and LAMP1 (*n* = 5). (**D**) Western blot analysis showed that TSA decreased the expression of ^Ser2448^p-mTOR, ^Thr389^p-S6K, and ^Ser757^p-ULK1, and increased the expressions of TFEB in nucleus compared with those of the model group (*n* = 5). All data are presented as mean ± SD. * *p* < 0.05 is significantly different as indicated, for values in the model group.

Transmission electron microscopy revealed that autolysosomes accumulated in the model group mice compared with the sham group mice, while TSA treatment reduced the accumulation of autolysosomes (Figure 3A). Additionally, we assessed lysosomal proteolysis by examining cathepsin B activity, with the results showing that cathepsin B activity was decreased in the whole hearts of mice treated with DOX, while TSA remarkably improved it (Figure 3B). The effects of TSA on autophagy-related proteins were further investigated by Western blot. The results showed that the levels of LC3-II and P62 were increased while the levels of Beclin1 and LAMP1 were reduced in the model group mice compared to those in the sham group, whereas TSA treatment attenuated the accumulation of LC3-II and P62 and upregulated the expression of Beclin1 and LAMP1 (Figure 3C). These results demonstrate that TSA may improve the lysosomal proteolytic process to ameliorate autolysosome accumulation by increasing the transcription and expression levels of LAMP1. We also investigated the upstream regulatory mTOR-ULK1/TFEB pathway. The Western blot results showed that the expression of ^Ser2448^p-mTOR in the model group mice was increased compared with that in the sham group mice. After treatment with TSA, the phosphorylated level of mTOR was decreased significantly (Figure 3D), indicating that TSA could restore autophagy via inhibiting the activation of mTOR. The expression of ^Thr389^p-S6K1 in the TSA-treated group was reduced, further confirming that the activation of mTOR was inhibited by TSA (Figure 3D). The activation of mTOR can directly inhibit autophagy by phosphorylating ULK1 at Ser757. The expression of ^Ser757^p-ULK1 was upregulated in the model group mice compared to that in the sham group mice, and TSA treatment was able to suppress the phosphorylation of ULK1 (Figure 3D). Furthermore, the expression of TFEB in the nucleus was significantly downregulated in the model group mice compared with that in the sham group mice, and TSA treatment upregulated the nuclear TFEB levels (Figure 3D). These results indicate that TSA was able to restore autophagy in the DIC mouse model. Pravastatin had similar effects to those of TSA on autophagy (Figure 3A–D).

### 2.4. DOX Inhibits Autophagic Flux and Lysosomal Proteolysis In Vitro

**Figure 4:**
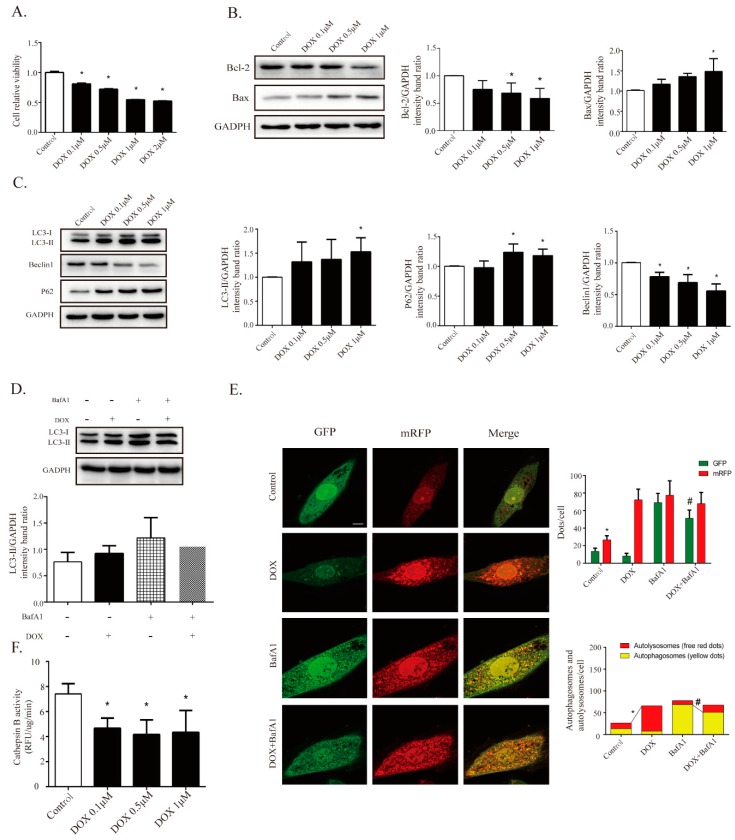
DOX is found to inhibit cardiomyocyte autophagic flux and lysosomal proteolysis in cardiomyocytes. (**A**) DOX decreased cell viability in a dose-dependent way via Cell Counting Kit-8 (CCK-8) assay (*n* = 12). Western blot results showed DOX regulated apoptosis-related proteins, including Bax (*n* = 5) and Bcl-2 (*n* = 5), as seen in (**B**), and autophagy-related proteins, including LC3-II (*n* = 5), P62 (*n* = 5), and Beclin1 (*n* = 5), as seen in (**C**). (**D**) BafA1 (100 nM, pre-treated for 4 h) treatment elicited a significant accumulation of LC3-II, and co-treatment with DOX diminished the effect of BafA1 (*n* = 5). (**E**) Cardiomyocytes were transfected with GFP-mRFP-LC3 adenovirus to detect autophagic flux with or without BafA1; the DOX-treated cells showed a decline in yellow puncta and an increase in red puncta compared with the control group (*n* = 30). Scale bar: 50 μm. (**F**) Measurement of cathepsin B activity in cardiomyocytes (*n* = 12). All cathepsin B activity measurements are represented as the change in RFU/min corrected to μg of protein. All data are presented as mean ± SD and Student’s *t*-test. * *p* < 0.05 is significantly different as indicated, for values in the control group. # *p* < 0.05 is significantly different as indicated, for values in the BafA1-treated group. BafA1, abbreviation of Bafilomycin A1.

To investigate the regulatory mechanism of TSA on autophagy, an in vitro DIC H9C2 cell model was established. The treatment of H9C2 cells with DOX for 24 h significantly decreased cell viability in a dose-dependent manner, as shown by the cell viability assay (Figure 4A). Western blot analysis showed that the level of Bax was significantly increased and that the level of Bcl-2 was decreased in H9C2 cells treated with DOX for 24 h compared to those in the control cells, indicating an elevated level of apoptosis (Figure 4B). To determine whether autophagy was involved in DIC, several key proteins in the autophagic signaling pathway were measured. The levels of LC3-II and P62 were significantly increased and the level of Beclin1 was decreased in the DOX treatment cells compared to those in the control cells (Figure 4C). Decreased cell viability might therefore result from a change in autophagic activity after treatment with DOX. A DOX-induced H9C2 cell injury model was established and a 1 μM DOX dose was applied to treat cells in vitro.

To explore whether the DOX-induced accumulation of LC3-II was due to enhanced autophagic flux or to impaired lysosomal degradation, the cells were incubated with bafilomycin A1 (BafA1), a classical inhibitor of the fusion of autophagosomes and lysosomes. BafA1 can inhibit the formation of autolysosomes and can cause the accumulation of autophagosomes. Hence, LC3-II could be viewed as a marker of autophagosome content after treatment with BafA1. The results showed that BafA1 (100 nM) treatment elicited a significant accumulation of LC3-II in the DMSO group, and cotreatment with DOX reduced LC3-II levels, suggesting that the formation of autophagosomes was inhibited by DOX (Figure 4D). In addition to Western blot analysis, H9C2 cells were transfected with GFP-mRFP-LC3 adenovirus and the LC3 contents were observed by fluorescence analysis. Increases in autophagic flux were indicated by an increase in yellow puncta (autophagosomes) and red puncta (autolysosomes). The DOX-treated cells showed a decline of yellow puncta, indicating decreased autophagosomal content (Figure 4E). Concomitantly, a robust increase in red puncta was observed in DOX-treated cells, indicating the accumulation of autolysosomes (Figure 4E). These results highlight that both autophagosome formation and autolysosomal degradation were impaired in DOX-treated cells.

The accumulation of autolysosomes induced by DOX could be due to the impaired function of lysosomal proteolysis. The activity of cathepsin B was therefore assessed. The results showed that cathepsin B activity was inhibited by DOX treatment in a dose-dependent manner in H9C2 cells (Figure 4F). These results further indicated that DOX suppressed lysosomal proteolytic activity and induced the accumulation of autolysosomes, then leading to retardation of autophagosome formation.

### 2.5. TSA Promotes Autophagosome Formation and Lysosomal Proteolysis

**Figure 5:**
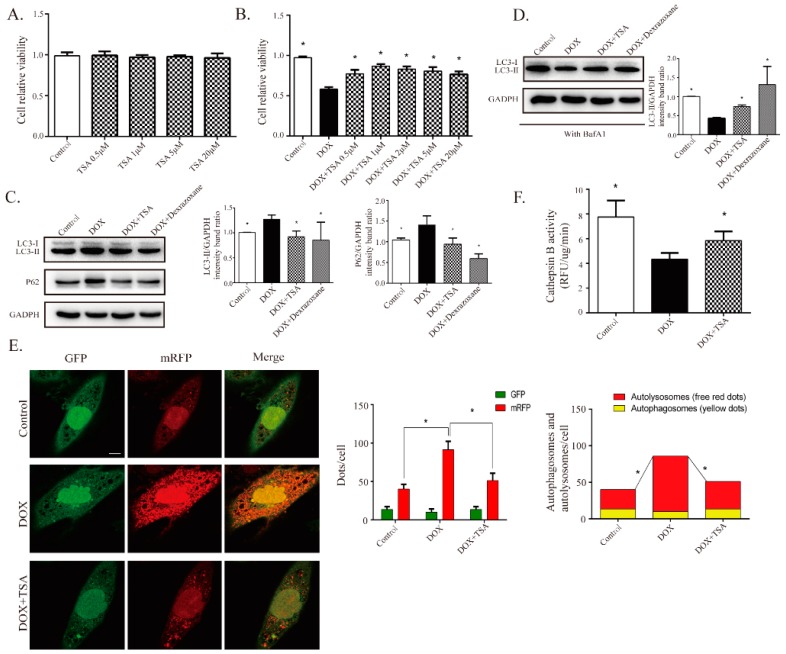
TSA is shown to promote autophagosome formation and lysosomal proteolysis. (**A**) TSA demonstrated no cytotoxic effect on cardiomyocytes below 10 μM (*n* = 12). (**B**) TSA attenuated DOX-induced cell injury, as evidenced by increased cell viability (*n* = 12). Western blot results showed that TSA can reverse the expressions of LC3-II (*n* = 5) and P62 without (*n* = 5) BafA1, as seen in (**C**), or reverse the expression of LC3-II with (*n* = 5) BafA1, as seen in (**D**). (**E**) Cardiomyocytes were transfected with GFP-mRFP-LC3 to monitor changes in autophagic flux. TSA increased autophagosomes (yellow puncta) and significantly reduced autolysosomes (red puncta) (*n* = 30). Scale bar: 50 μm. (**F**) Measurement of cathepsin B activity in each group (*n* = 12). * *p* < 0.05 is significantly different as indicated, for values in the DOX group.

After exploring the dysregulation of autophagic flux by DOX in H9C2 cells, the effects of TSA on DOX-stimulated H9C2 cells were investigated. Cell Counting Kit-8 (CCK-8) assaying showed that the cotreatment of H9C2 cells with TSA significantly increased cell viability (Figure 5A,B).

The effects of TSA on autophagic flux were further studied in vitro. As shown in Figure 5C, DOX-induced increases in LC3-II and P62 were alleviated by TSA, suggesting that TSA played a role in regulating the autophagy that was impaired by DOX. After blocking the formation of autolysosomes by BafA1, the LC3-II content was reduced by DOX, and treatment with TSA upregulated the LC3-II levels in DOX-stimulated cells, indicating that TSA could promote the formation of autophagosomes (Figure 5D). Dexrazoxane, the positive control, showed a similar effect to that of TSA (Figure 5C,D). A previous study has reported that dexrazoxane has been observed to regulate autophagy in a DIC model [22]. Furthermore, fluorescence analysis showed that TSA was able to increase the contents of autophagosomes and decrease the contents of autolysosomes in DOX-stimulated cells (Figure 5E). The activity of cathepsin B in autolysosomes was also increased by TSA (Figure 5F), indicating that TSA can activate lysosomal proteolysis and promote the degradation of autolysosomes.

### 2.6. TSA Enhances Autophagic Flux by Regulating the Beclin1/LAMP1 Signaling Pathway

**Figure 6:**
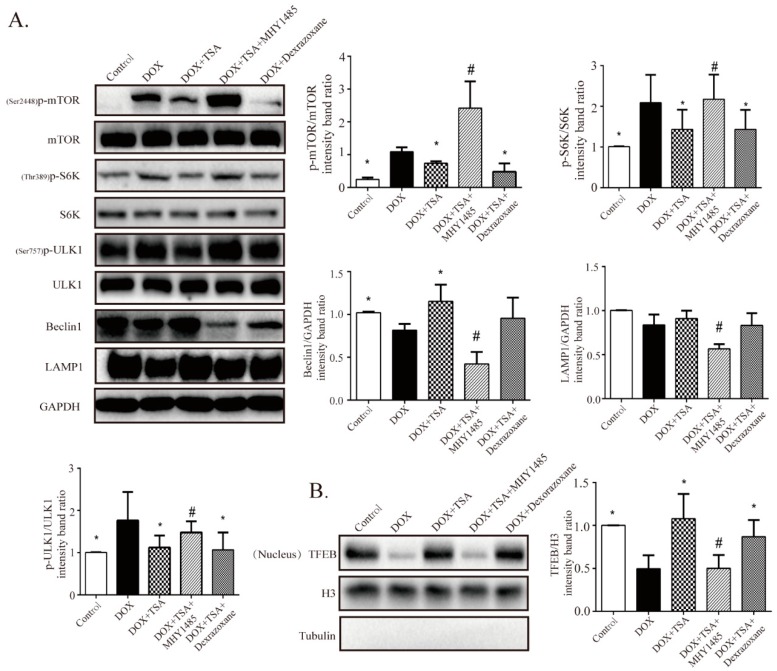

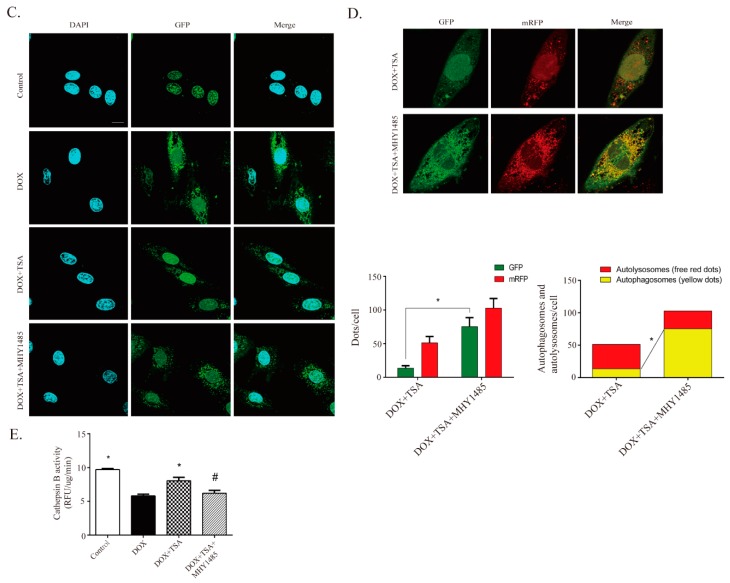
TSA is observed to activate autophagic flux via the Beclin1/LAMP1 pathway. (**A**,**B**) Western blot results showed TSA activated the ULK1-Beclin1/TFEB-LAMP1 cascade pathway, thereby promoting autophagy in cardiomyocytes. After MHY1485 treatment, the effect of TSA on autophagy-related proteins was inhibited (*n* = 5). (**C**) Cardiomyocytes were transfected with GFP-TFEB to monitor the cytoplasm-location of TFEB. Results showed TSA decreased the phospho-cytoplasm recruitment of TFEB and increased its nuclear localization by mTOR (*n* = 30). Scale bar: 50 μm. (**D**) After MHY1485 treatment, the effect of TSA on autophagic flux was inhibited (*n* = 30). Scale bar: 50 μm. (**E**) After MHY1485 treatment, the effect of TSA on cathepsin B activity was abolished (*n* = 12). All data are presented as mean ± SD. * *p* < 0.05 is significantly different as indicated, for values in the model group. # *p* < 0.05 is significantly different as indicated, for values in the TSA-treated group.

The effects of TSA on the Beclin1/LAMP1 signaling pathway were further investigated. Consistent with in vivo experiments, Western blot results demonstrated that phosphorylation of mTOR, S6K1, and ULK1 were upregulated and that Beclin1 and LAMP1 were downregulated in the model group mice compared to the control group mice (Figure 6A). Dramatically, TSA treatment was able to reverse these changes (Figure 6A). These results indicate that TSA may promote the formation of autophagosomes and the degradation of autophagosomes by targeting Beclin1/LAMP1. We also found that TSA was able to upregulate nuclear TFEB content (Figure 6B). To further investigate the effects of TSA on TFEB recruitment, H9C2 cells were transiently transfected with a lentiviral vector carrying TFEB-GFP. The results showed that DOX increased the cytoplasmic recruitment of TFEB and suppressed its nuclear localization, while in the TSA-treated group, the cytoplasmic localization of TFEB was reduced and the nuclear recruitment of TFEB was upregulated (Figure 6C).

To further confirm that TSA promoted autophagy through the Beclin1/LAMP1 signaling pathway, the mTOR agonist MHY1485 was utilized in this study. Coincubation with MHY1485 abrogated the protective effects of TSA on autophagic flux (Figure 6D). Western blot analysis showed that the effects of TSA on the expression of ^Ser2448^p-mTOR, ^Thr389^p-S6K1, ^Ser757^p-ULK1, Beclin1, LAMP1 and TFEB were all abolished by the mTOR agonist (Figure 6A,B). In addition, MHY1485 could also abolish the effect of TSA on TFEB localization and cathepsin B activity (Figure 6C,E), further demonstrating that the effects of TSA on autophagy were mediated by ULK1-Beclin1/TFEB-LAMP signaling pathway.

### 2.7. TSA Reduces Cardiotoxicity without Compromising the Antitumor Activity of DOX

**Figure 7:**
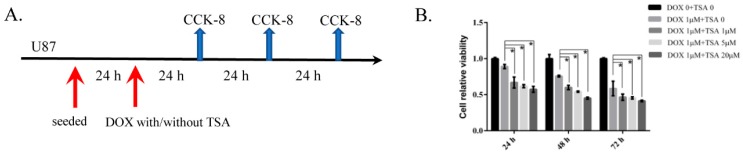
Proliferative inhibition assay of DOX and TSA in combination with DOX on U87 human glioblastoma cell lines. (**A**) Cells were exposed to DOX in combination with/or without various concentrations of TSA for 24 h/48 h/72 h. (**B**) Cell viability curves were accessed by CCK-8 assay. * *p* < 0.05.

CCK-8 results showed that compared to individual DOX treatment, co-treatment with TSA in different doses did not attenuate the effect of DOX on tumor cells, which indicates that TSA was able to reduce the side effects of DOX on the heart without affecting its anti-tumor effects (Figure 7B).

## 3. Discussion

TSA has been shown to have potential cardioprotective effects against DIC [19,20,23,24,25]. However, the protective mechanisms of TSA remain to be fully understood. In this study, we have explored the regulatory effects of TSA on autophagy by in vivo and in vitro studies. The main findings are as follows: (1) TSA was shown to improve heart function and reverse structural alterations in a DIC zebrafish model; (2) TSA was observed to significantly improve cardiac function in a DIC mouse model by inhibiting apoptosis and restoring autophagy; (3) DOX was shown to disturb the dynamic balance of autophagosomes/autolysosomes and downregulate autophagic flu; (4) TSA was observed to restore autophagic flux and improve the cell viability of DOX-stimulated H9C2 cells via increasing autophagosome formation and autolysosome degradation; (4) the efficacy of improving autophagic flux was shown to be mediated by the Beclin1/LAMP1 pathway.

DOX is an effective anticancer chemotherapeutic agent used for the treatment of hematologic malignancies and solid tumors. However, prolonged use of DOX results in acute and chronic cardiotoxicity, which accelerates the process of heart failure. Autophagy is a cell survival process whereby cells degrade damaged, malformed, and long-lived proteins within the lysosome to maintain cardiac homeostasis [26]. Previous reports have demonstrated that the dysregulation of autophagy may contribute to DIC [1,7,27]. However, there are conflicting findings in addressing the possible contribution of autophagic flux in DIC. Autophagy has been reported to be either decreased or increased in different studies [27,28,29,30,31,32,33]. Several factors have accounted for the discrepancies in these studies. First, a large number of studies have been based on models with short-term and high-dose DOX exposure which do not accurately reflect the clinical scenario of chronic DIC. To address this issue, we established a model that involved multiple intravenous injections of DOX at clinically used doses. Second, many studies have focused on just one stage of autophagy instead of comprehensively evaluating autophagy as a process of flux. In fact, different stages of autophagy may exhibit different characteristics. Caution has been recommended by prior studies due to the observed results that increases in the LC3-II level may result from the excess generation of autophagosomes and may also be due to the inefficient clearance of autolysosomes, which could be an effect of blocked autophagosome-lysosome fusion and/or autolysosome degradation (decreased lysosomal function) [1,26]. Moreover, the dysregulation of the autophagy-lysosomal system results in the accumulation of P62. Therefore, an increase in LC3-II and P62 does not necessarily mean that autophagy is upregulated. It is important to assess autophagic flux as a whole process. In this study we have comprehensively evaluated the effects of DOX on changes in autophagic flux in DOX-treated myocardial H9C2 cells. The levels of LC3-II and P62 were seen to increase and the level of Beclin1 reduced; this is consistent with results from the DIC mouse model. We further examined the effects of DOX on autophagic flux by blocking autophagosome–lysosome fusion with BafA1. After blocking the formation of autolysosomes by BafA1, LC3-II was able to be used as a marker for the contents of autophagosomes [1]. The LC3-II levels in DOX-treated cells were reduced in response to BafA1 cotreatment, indicating that DOX could reduce the contents of autophagosomes. Moreover, by applying the fluorescent GFP-mRFP-LC3 reporter system, we also observed an accumulation of undegraded autolysosomes in DOX-treated H9C2 cells, suggesting that DOX could impair the function of autolysosomes. At present, biogenesis of autophagosomes in the early stage of autophagy is relatively well explored, whereas the molecular mechanisms controlling the later phrase of autophagy, including regulation of lysosomal function and autolysosome degradation, need to be fully understood. Prior studies have revealed that DOX is able to inhibit lysosomal acidification and suppress lysosomal function [27]. Our data further confirmed that DOX perturbed the function of autolysosomes via the inhibition of lysosomal proteolysis, as the activity of cathepsin B was shown to be suppressed by DOX. The above findings demonstrated that DOX downregulated autophagic flux, which was characterized by suppressing the formation of autophagosomes as well as the degradation of autolysosomes. On this basis, there are reasons to believe that the amount of autophagosomes and autolysosomes always holds the dynamic balance as usual and that interruption of autolysosome degradation will result in accumulation of autolysosomes, which further retards autophagosome formation. It is necessary to find a signaling pathway that regulates autophagosome synthesis, maturation, clearance, and lysosomal biogenesis [10,34,35].

TSA is an extract from *Salvia miltiorrhiza* Bge that has been shown to have a cardioprotective effect [17,18,36]. Studies have shown that it can reverse changes in heart rate and shorten the ST and QRS intervals prolonged by DOX [20]. The results of our study demonstrated that TSA treatment protected heart function in a DIC zebrafish model and improved cardiac function in a DIC mouse model. Transmission electron microscopy indicated that TSA treatment reduced the accumulation of autolysosomes. We further investigated the effects and underlying mechanisms of TSA on the autophagy pathway. The levels of LC3-II and P62 were reduced by TSA treatment, suggesting that TSA is able to promote the clearance of autolysosomes, which was suppressed by DOX treatment. The results of the in vitro studies showed that TSA was able to reduce the contents of autolysosomes and increase the activity of cathepsin B in DOX-stimulated H9C2 cells, confirming that TSA was able to promote the degradation of autolysosomes. Levels of Beclin1 and the contents of autophagosomes were increased in both the in vivo and in vitro studies, demonstrating that TSA was able to upregulate the formation of autophagosomes. Taken together, this means that TSA can ameliorate DIC and can improve autophagic flux by promoting autophagosome formation and autolysosome degradation. In addition, we also found that TSA was able to reduce apoptosis in the DIC model. Autophagy has been shown to engage in complex interplay with apoptosis under different situations [37,38]. In the DIC model, the dysregulation of autophagy may contribute to cellular apoptosis, which further aggravates cardiac injury, and TSA protects against injury by restoring autophagy and by suppressing apoptosis.

The effects of TSA on the upstream regulatory autophagic pathway were further investigated. TFEB is a transcriptional regulator that drives the expression of autophagy and lysosomal genes such as LAMP1 [39,40,41,42]. The transcriptional effects of TFEB are inhibited by the phosphorylation of mTOR, leading to the sequestration of TFEB in the cytoplasm. mTOR also induces the inhibitory phosphorylation of ULK1, thereby inhibiting Beclin1-mediated autophagosomal initiation [5,43]. Taken together, the Beclin1/LAMP1 pathway plays a vital role in keeping the amount of autophagosomes and autolysosomes in dynamic balance via regulating autophagosome synthesis, maturation, clearance, and lysosomal biogenesis. Consistent with the results of previous reports, our study showed that DOX dysregulated the expression of Beclin1, LAMP1, and the upstream targets in the autophagy pathway [1,27,29]. The expression of mTOR was increased by DOX, thereby restricting TFEB in the cytoplasm and inhibiting the transcription of autophagy-related genes. Treatment with TSA inhibited mTOR activity and promoted the nuclear localization of TFEB, where it could activate the expression of LAMP. In addition, TSA weakened the inhibitory phosphorylation on ULK1, thereby activating the Belcin1-mediated autophagosomal process. To further confirm the inhibitory effect of TSA on Beclin1/LAMP1, MHY1485, which is an agonist of mTOR, was applied to investigate whether MHY1485 could abolish the protective effects of TSA. The results showed that after coincubation with MHY1485, the effects of TSA on autophagic flux and autophagy-related proteins, including mTOR, TFEB, S6K, ULK1, Beclin1, and LAMP1, were abrogated in DOX-stimulated H9C2 cells. In addition, the cellular protective effects of TSA were also abrogated by MHY1485, suggesting that the regulatory effects of TSA on autophagy were mainly mediated through the Beclin1/LAMP1 pathway. The Belcin1/LAMP1 pathway may serve as a novel target in the treatment of DIC.

## 4. Materials and Methods

### 4.1. Establishment of a DIC Model in Zebrafish

A DIC model in zebrafish was induced according to the methods of a previous study [44]. Briefly, zebrafish were treated by DOX or/and TSA one day postfertilization (dpf) after the heart had formed and circulation had begun. Then, 100 μM DOX and 20 μM TSA were used and phenotypic changes, including FS, heart rate, the blood speed of the tail vein, and survival rate, were assessed at three dpf.

### 4.2. Establishment of a DIC Model in Mice

C57BL/6 mice (20 g ± 2 g) were purchased from Beijing SPF Biotechnology Co., Ltd., Beijing, China. The protocol of “Guiding Principles in the Use of Animals in Toxicology” was followed. The mice were randomized into four groups. There were sixteen mice in each of the model groups, the TSA-treated group, and the positive drug (pravastatin)-treated group, and ten mice in the sham group. For mice in the model, TSA-treated, and pravastatin-treated groups (*n* = 16 per group), DIC was induced via tail vein injection with DOX (5 mg·kg^−1^) once per week for four consecutive weeks. This mouse model has been in use for several years [1,20]. For animals in the sham group (*n* = 10), saline was given without treatment of DOX. One week after four injections, each drug was orally administered for 4 weeks, and mice in the sham group and model group received the same volume of saline solution. The doses of TSA (10 mg·kg^−1^) and pravastatin (40 mg·kg^−1^) in our study were set based on a previous report [45,46]. All animals were maintained in a 12 h light/dark cycle from six AM to six PM at a temperature of 22 ± 2 °C and were allowed free access to food and water. During the treatment period, the body weights of the mice were recorded every week. All experimental procedures and animal care were approved by the Beijing University Chinese Medicine Animal Care Committee (approval code: BUCM-4-2018101504-4068) and were carried out in accordance with British Journal of pharmacology (BJP) Policy [47].

### 4.3. Echocardiographic Assessment of Cardiac Functions

The cardiac function of the left ventricle was examined by echocardiography (Vevo TM 2100; Visual Sonics, Toronto, ON, Canada). LVEDD, LVESD, left ventricular end-diastolic volume (LVEDV), left ventricular end of systolic volume (LVESV), EF and FS were assessed for at least three uninterrupted cardiac cycles. EF% = [(LVEDV − LVESV)/LVEDV] × 100%. FS% = [(LVED; d − LVEDs)/LVED; d] × 100%.

### 4.4. Measurement of LDH and CKMB

Blood was taken into collection tubes and was allowed to stand for two hours at room temperature. Then, blood samples were centrifuged for 15 min at 3000 r/min. The upper serum was transferred into new 1.5 mL Eppendorf tubes for the detection of LDH and CKMB.

### 4.5. Histological Examination and Masson Staining

Cardiac tissues were immersed in 4% paraformaldehyde for at least 24 h and then embedded in paraffin and sectioned into 4 μM serial sections. The paraffin sections were stained with H&E. Masson’s trichrome stain was assessed to detect myocardial fibrosis. Finally, the images were visualized under an optical microscope at 400× magnification.

### 4.6. TUNEL Staining

The level of cardiac apoptosis in mice was detected by TUNEL staining following the manufacturer’s specifications (Roche, Basel, Switzerland). Images were randomly selected and analyzed.

### 4.7. Electron Microscopy

The cardiac tissues were immersed in 4% paraformaldehyde for two hours at room temperature and were then fixed with 2.5% (*v*/*v*) glutaraldehyde in 0.1 mol/L sodium cacodylate buffer at 4 °C overnight. Afterwards, specimens were washed three times with 0.1 mol/L sodium cacodylate buffer and then fixed in 1% osmium tetroxide and 0.8% K_3_[Fe(CN_6_)] in 0.1 mol/L sodium cacodylate buffer for one hour at room temperature. After washing three times with water, the specimens were dehydrated with increasing concentrations of ethanol and were infiltrated with Embed-812 resin; finally, the specimens were polymerized in a 60 °C oven overnight. The blocks were sectioned with a diamond knife, placed onto copper grids and poststained with 2% uranyl acetate in lead citrate and water. The images were acquired by observation under an electron microscope (Leica, Buffalo Grove, IL, USA).

### 4.8. Western Blot Analysis

Myocardial tissues or cells were lysed with pre-cooled RIPA lysate (Beijing Applygen Technology Inc., Beijing, China) with 1% protease inhibitor and 2% protein phosphatase inhibitor (Beijing Applygen Technology Inc.). A bicinchoninic acid (BCA) protein assay kit (Beijing Applygen Technology Inc.) was used to measure the protein concentration. To determine the expression of related proteins, 50 mg protein of each sample was loaded onto 10% sodium dodecyl sulfate polyacrylamide gel electrophoresis (SDS-PAGE) gels and was transferred onto a polyvinylidene fluoride (PVDF) membrane, which was blocked with 5% skim milk in 1× Tris-buffered saline for two hours. The membranes were incubated with primary antibodies at 4 °C overnight and with the secondary antibody at room temperature for one hour; then, the membranes were exposed to ECL (ECL Plus Western blotting Detection Reagent, GE Healthcare, Fairfield, CT, USA) in a darkroom for 1 min. The antibodies we used were as follows: anti-mTOR (Cell Signaling Technology, Boston, MA, USA, Cat# 2983, RRID:AB_2105622), anti-phospho mTOR (Cell Signaling Technology, Cat# 2971, RRID:AB_330970), anti-ULK1 (Cell Signaling Technology, Cat# 8054, RRID:AB_11178668), anti-phospho ULK1 (Cell Signaling Technology, Cat# 14202, RRID:AB_2665508), anti-Beclin 1 (ab62557; Abcam, Cambridge, UK), anti-S6K1 (ab9366; Abcam), anti-LC3 (ab128025; Abcam), anti-phospho S6K1 (ab2571; Abcam), anti-p62 (ab56416; Abcam), anti-lysosomal-associated membrane protein (LAMP)1 (ab24170; Abcam), anti-GAPDH (ab8245; Abcam), anti-Bcl-2 (ab7973; Abcam), anti-Bax (ab32503; Abcam), anti-rabbit IgG H&L (ab16284; HRP; Abcam), anti-mouse IgG H&L (ab97250; HRP; Abcam), anti-tubulin (ab59680; Abcam), anti-histone H3 (ab1791; Abcam), and anti-TFEB (13372-1-AP; Proteintech, Rosemont, IL, USA). The original western blot pictures were shown in the Supplementary Materials (Figures S1–S5).

### 4.9. Establishment of DOX-Induced H9C2 Cell Injury Model and Cell Viability

H9C2 cells were cultured dulbecco’s modified eagle medium (DMEM) containing 10% fetal bovine serum (FBS) in a 37 °C humidified atmosphere containing 5% CO_2_ and 95% O_2_. The cells were seeded onto 96-well plates at a density of 6 × 10^4^ cells/mL for 24 h before being treated with different concentrations of DOX (0.1~2 μmol/L). It has been suggested that DOX concentrations > 2 μmol/L do not reflect the clinically relevant context, so we used DOX below 2 μmol/L [1]. After 24 h of treatment with DOX, the cell viability was detected using a Cell Counting Kit-8 (CCK-8, Dojindo, Kumamoto, Japan). The original medium was discarded and 10% CCK-8 in DMEM solution was added to each well for two hours of incubation at 37 °C. Finally, the absorbance was measured at 450 nm using a Microplate reader (Perkin-Elmer, Waltham, MA, USA). In addition, H9C2 cells (6000 cells/well) were cultured in 96-well microtiter plates. After incubation with TSA (0.5~20 μM) for 24 h, cells were cotreated with 1 µM DOX for 24 h. Subsequently, CCK-8 was used to assess cellular viability. H9C2 cells were divided into a control group, model group (with 1 μM DOX), TSA-treated group (with 1 μM TSA), TSA+MHY1485-treated group (with 5 μM MHY1485 coincubation), and dexrazoxane-treated group (with 10 μM dexrazoxane, ten times the clinically recommended dose of DOX). H9C2 cells (6000 cells/well) were cultured in 96-well microtiter plates. After incubation with 1 μM TSA with or without MHY1485 for 24 h, the cells were treated with 1 µM DOX for 24 h. MHY1485 is an agonist of mTOR. Dexrazoxane was applied as the positive drug. Dexrazoxane is the only FDA-approved agent for the prevention of DIC, and the recommended dose is 10 times that of DOX [48].

### 4.10. Cell Viability Analysis for U87 Cells

To assess viability of U87 cells, cells (3000 cells/well) were seeded in 96 wells. After 24 h, cells were exposed to DOX in combination with/or without various concentrations (1 μM, 5 μM, and 20 μM) of TSA for 24 h/48 h/72 h (Figure 7A). Viable cells were counted in each well.

### 4.11. Assessment of Apoptosis by a Hoechst 33258 Staining Kit

H9C2 cells were washed with 0.1 mol/L sodium cacodylate buffer and were fixed with ice-cold 4% paraformaldehyde for 20 min. Afterwards, the cells were washed three times with 0.1 mol/L sodium cacodylate buffer and were stained with Hoechst 33258 (Beijing Solarbio Science & Technology Co., Ltd., Beijing, China) for 15 min in the dark at 37 °C. Finally, the samples were visualized under an inverted fluorescence microscope (Olympus; BX50-FLA; Tokyo, Japan).

### 4.12. Cathepsin B Activity Assay

After the treatments, a cathepsin B activity assay kit (ab65300, Abcam) was utilized. The cells/tissues were harvested and washed with cold 0.1 mol/L sodium cacodylate buffer in different groups. The cells were then resuspended in chilled cell lysis buffer on ice for 30 min. After centrifugation at the top speed, 50 μg cell lysate was prepared. The CB reaction buffer and CB substrate were added to each well. After incubation at 37 °C for two hours protected from light, the samples were measured at 400/505 nm on a fluorescence microplate.

### 4.13. GFP-mRFP-LC3 Adenovirus Transfection

H9C2 cells were transduced with GFP-mRFP-LC3 adenovirus (purchased from Hanbio Technology, Shanghai, China) for 8 h at a concentration of multiplicity of infection (MOI) = 10 according to the supplier’s protocol. After 24 h, the cells were treated as designated. The cells were observed under a confocal microscope. The GFP-mRFP-LC3 makes it possible to distinguish between autophagosomes and autolysosomes: the acid-sensitive GFP fluorescence will quench after the autophagosomes fuse with the lysosomes as the pH decreases, while the acid-insensitive mRFP will not be lost in either the autophagosomes or autolysosomes. The colocalization efficiency of GFP with mRFP signals was measured using ImageJ software (https://imagej.nih.gov/ij/).

### 4.14. GFP-TFEB Lentiviral Vector Transfection

TFEB was overexpressed by transfecting a lentiviral vector carrying GFP-TFEB purchased from Hanbio Technology (Shanghai, China). The transduction lasted for 24 h at a concentration of MOI = 30. The antibiotic-resistant transfected H9C2 cells were selected using 1 μg/mL puromycin. To observe the subcellular localization of GFP-TFEB, 4% paraformaldehyde was used to fix the cells at room temperature for 20 min. The samples were then washed three times with 0.1 mol/L sodium cacodylate buffer and were incubated with DAPI (Beijing BioDee Biotechnology Co., Ltd., Beijing, China) for 20 min at 37 °C. Finally, the cells were observed under an inverted fluorescence microscope.

### 4.15. Data and Statistical Analysis

Statistical analyses were performed using GraphPad Prism software 6.0 (San Diego, CA, USA). All results have been presented as mean ± SD. For statistical analysis, one-way ANOVA was performed following Dunnett’s post hoc tests. Comparisons between the two groups were analyzed by Student’s *t*-test. The difference was considered statistically significant when *p* < 0.05. The statistical analysis abided the recommendations of the experimental design and analysis in pharmacology [47].

### 4.16. Materials

Saline was purchased from SiYao Co., Ltd. (Shijiazhuang, China). Doxorubicin was purchased from ApexBio Technology LLC (Houston, TX, USA). Tanshinone IIA was purchased from Shanghai Shidande Standard Technical Service Co., Ltd. (Shanghai, China). Pravastatin was purchased from Sangong Pharmaceutical (Shanghai) Co., Ltd. (Shanghai, China). 4% paraformaldehyde was from Beijing Applygen Technology Inc. DMSO was purchased from Sigma-Aldrich LLC (Shanghai, China). Dexrazoxane was purchased from Liaoning Norvino Pharmaceutical Co., Ltd. (Liaoning, China) MHY1485 was purchased from Selleck (Houston, TX, USA). DMEM, FBS, and 0.1 mol/L sodium cacodylate buffer were purchased from Beijing BioDee Biotechnology Co., Ltd. (Beijing, China). GFP-mRFP-LC3 adenovirus and GFP-TFEB lentiviral vector were purchased from Hanbio Technology Co., Ltd. (Shanghai, China). All other chemicals were purchased from commercial sources and were maintained at the highest purity available.

## 5. Conclusions

In conclusion, in this work TSA was shown to be able to exert a cardioprotective effect against DIC by restoring the dynamic balance of autophagosomes and autolysosomes. The protective effect mainly manifested in upregulating the autophagic flux by improving autophagosome formation and autolysosome clearance via the Beclin1/LAMP1-mediated pathway. Our study provides new insight into the pharmacological mechanism of TSA and offers a potential therapeutic strategy for the management of DIC in the clinic. In addition, as a novel agent in postoperative adjuvant therapy combined with DOX, TSA can improve the sensibility of DOX for gliomas with fewer cardiotoxicity. All in all, this study builds a platform for evaluating and developing the antitumor effects of Chinese herbal medicines in combination with chemotherapeutic drugs.

## Supplementary Materials

The following are available online at https://www.mdpi.com/2072-6694/11/7/910/s1, Figure S1: Panel 1 represents western blot shown in Figure 2E, Figure S2: Panel 1 represents western blot shown in Figure 3C,D, Figure S3: Panel 1 represents western blot shown in Figure 4B–D; Figure S4: Panel 1 represents western blot shown in Figure 5C,D, Figure S5: Panel 1 represents western blot shown in Figure 6A,B.
