# Supplementary material for: Tanshinone IIA Restores Dynamic Balance of Autophagosome/Autolysosome in Doxorubicin-Induced Cardiotoxicity via Targeting Beclin1/LAMP1
Source: Cancers (Basel). 2019 Jun 28;11(7):910. doi: 10.3390/cancers11070910 (PMC6679133; doi:10.3390/cancers11070910)

Figure S1: Panel 1 represents western blot shown in Figure 2E.

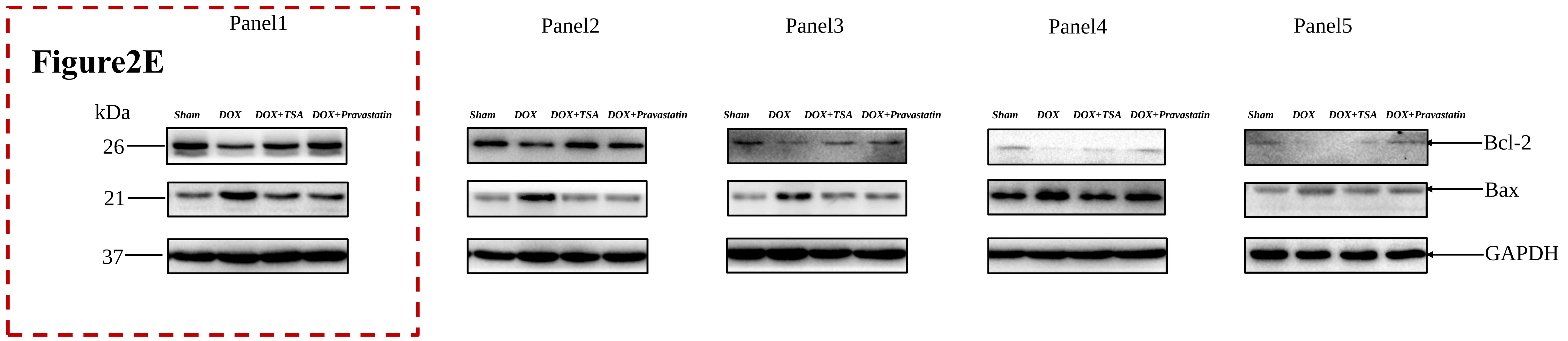

Figure S2: Panel 1 represents western blot shown in Figure 3C,D.

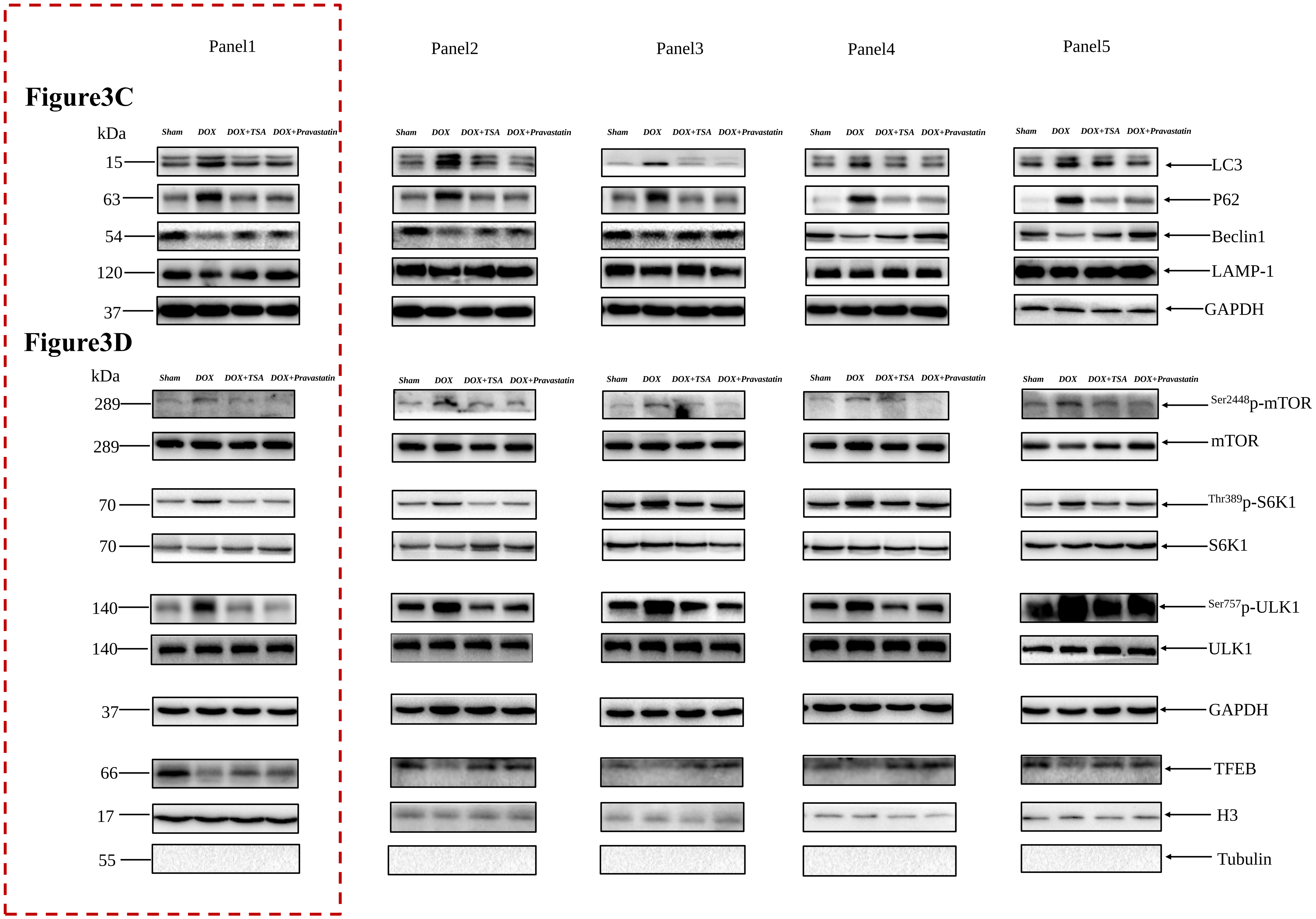

Figure S3: Panel 1 represents western blot shown in Figure 4B-D.

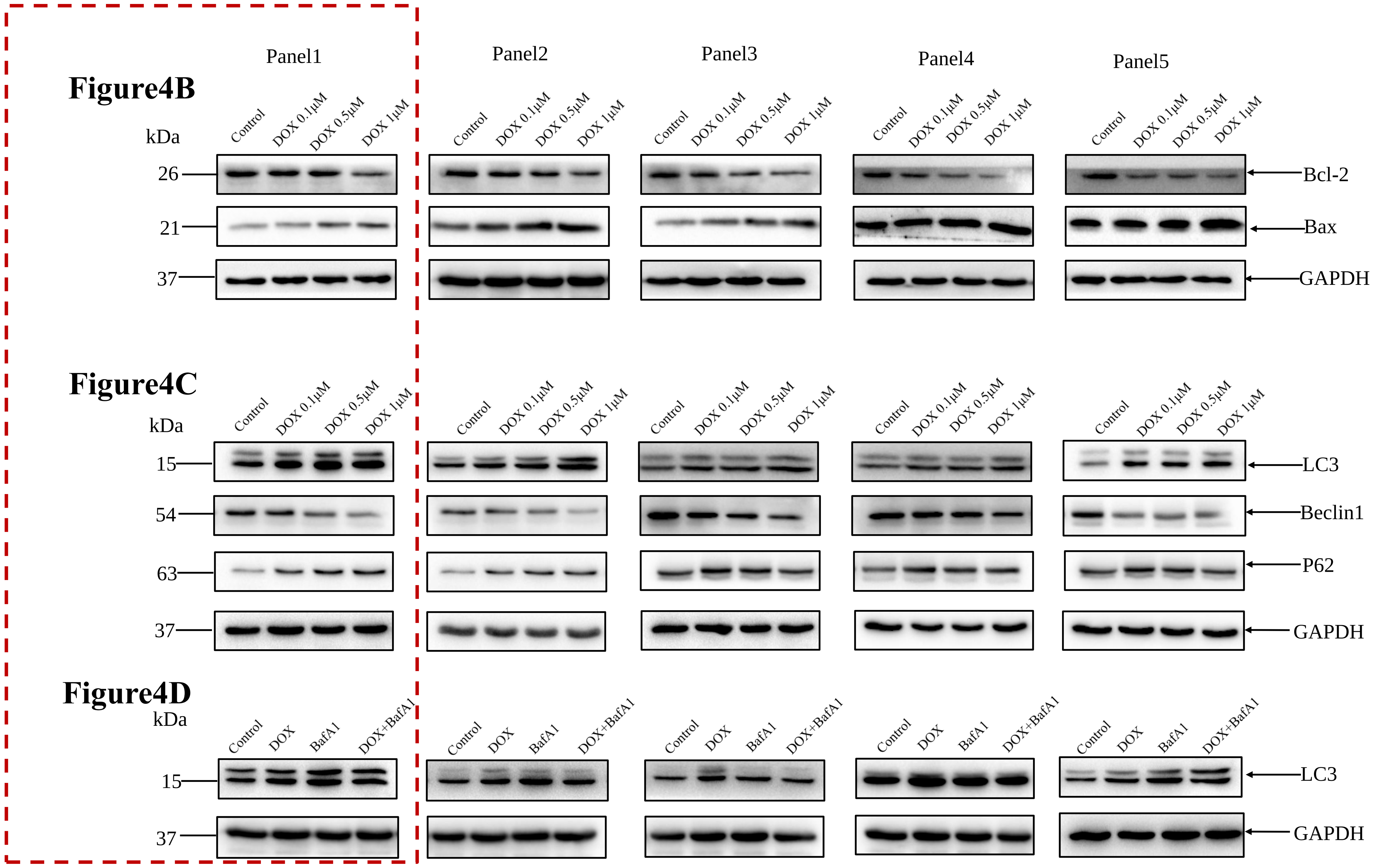

Figure S4: Panel 1 represents western blot shown in supplement Figure 5C,D.

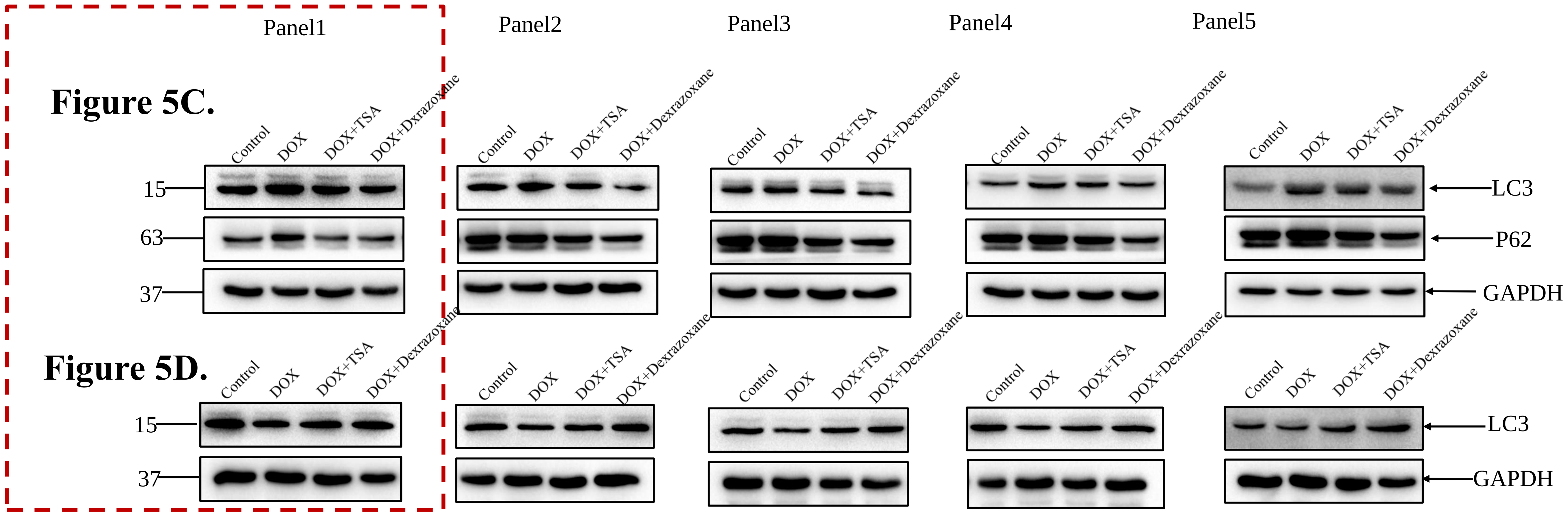

Figure S5: Panel 1 represents western blot shown in Figure 6A,B

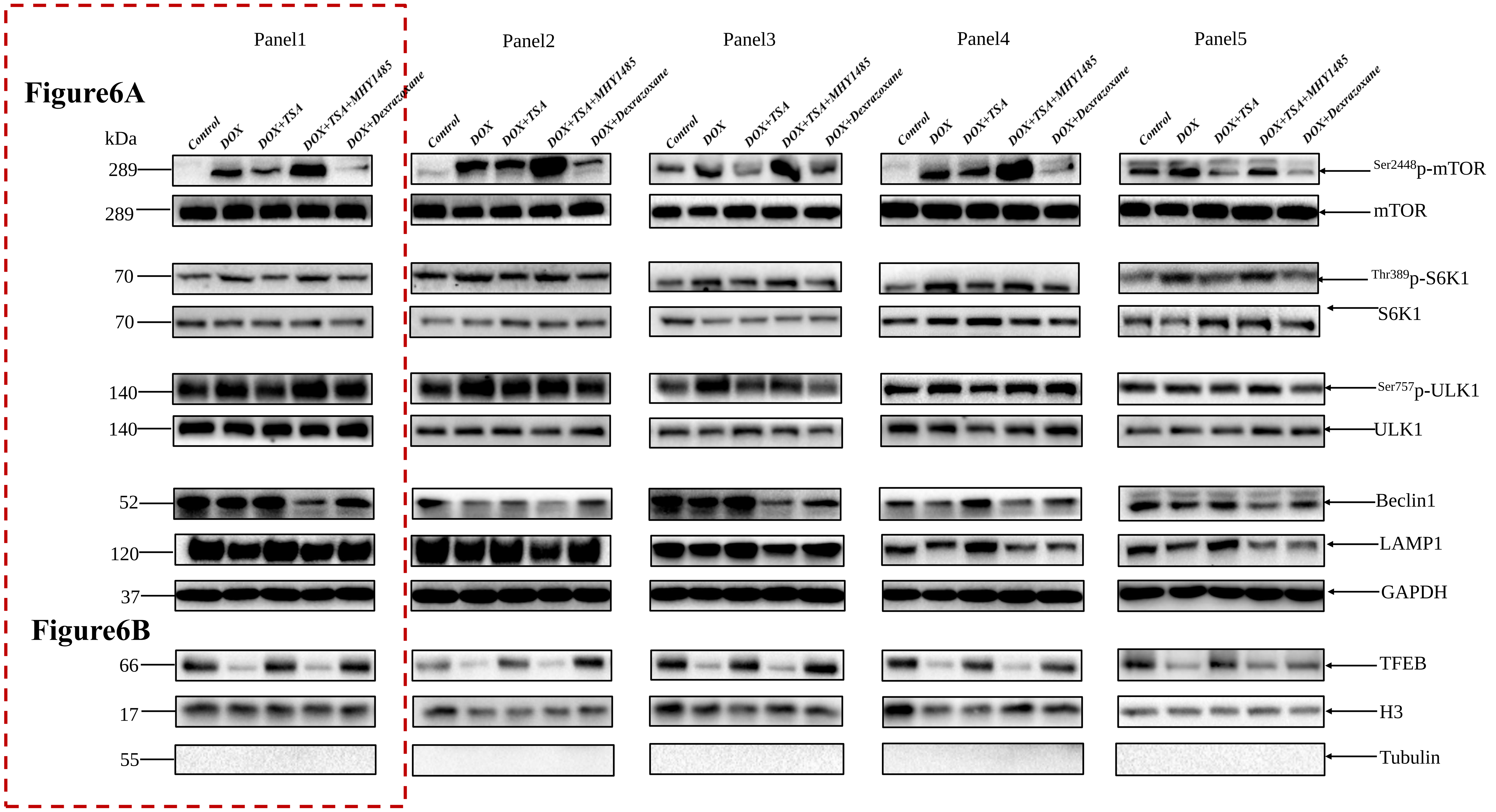

Supplement: Supplementary file 1 [file cancers-11-00910-s001.pdf]
